# Gut microbiota and functional outcome after ischemic stroke: a Mendelian randomization study

**DOI:** 10.3389/fimmu.2024.1414653

**Published:** 2024-09-23

**Authors:** Dian Qu, Deming Jiang, Yan Xin, Guichun Yang, Huan Liang, Linlin Wang

**Affiliations:** ^1^ Department of Neurology, Harbin 242 Hospital, Harbin, Heilongjiang, China; ^2^ Department of Neurology, Xuanwu Hospital, Capital Medical University, Beijing, China; ^3^ Department of Scientific Research, Harbin 242 Hospital, Harbin, Heilongjiang, China; ^4^ Department of Clinical Laboratory, Harbin Medical University Cancer Hospital, Harbin, Heilongjiang, China

**Keywords:** gut microbiota, ischemic stroke, functional outcome, synapse function, Mendelian randomization

## Abstract

**Background:**

Previous studies have shown that gut microbiota dysbiosis could affect clinical prognosis through an unknown mechanism. However, the causal relationship between the gut microbiota and the functional outcome after ischemic stroke remains unclear. We aimed to investigate the causal association between the gut microbiota and the functional outcome after ischemic stroke using Mendelian randomization (MR).

**Methods:**

Genetic instrumental variables associated with 211 bacterial traits were obtained from the MiBioGen consortium (*N* = 18,340). Data from genome-wide association studies (GWAS) for functional outcome after ischemic stroke were obtained from two phenotypes (i.e., overall stroke outcome and motor recovery). The inverse variance weighted method was used to estimate the causal association. Enrichment analysis was conducted based on the results of the MR analyses.

**Results:**

The genetically predicted family Peptostreptococcaceae (OR = 0.63, 95% CI = 0.41–0.98, *p* = 0.038) and the genera *LachnospiraceaeNK4A136 group* (OR = 0.65, 95% CI = 0.43–1.00, *p* = 0.048), *LachnospiraceaeUCG004* (OR = 0.54, 95% CI = 0.33–0.90, *p* = 0.017), and *Odoribacter* (OR = 0.40, 95% CI = 0.21–0.77, *p* = 0.006) presented a suggestive association with favorable functional outcome, while the genera *Eubacterium oxidoreducens group* (OR = 1.77, 95% CI = 1.11–2.84, *p* = 0.018) and *RuminococcaceaeUCG005* (OR = 1.85, 95% CI = 1.15–2.96, *p* = 0.010) were associated with unfavorable functional outcome. The genetically predicted family Oxalobacteraceae (OR = 2.12, 95% CI = 1.10–4.11, *p* = 0.025) and the genus *RuminococcaceaeUCG014* (OR = 4.17, 95% CI = 1.29–13.52, *p* = 0.017) showed a suggestive association with motor recovery, while the order Enterobacteriales (OR = 0.14, 95% CI = 0.02–0.87, *p* = 0.035) and the family Enterobacteriaceae (OR = 0.14, 95% CI = 0.02–0.87, *p* = 0.035) were associated with motor weakness. Enrichment analysis revealed that regulation of the synapse structure or activity may be involved in the effect of the gut microbiota on the functional outcome after ischemic stroke.

**Conclusions:**

This study provides genetic support that the gut microbiota, especially those associated with short-chain fatty acids, could affect stroke prognosis by mediating synapse function. Our findings suggest that modifying the composition of the gut microbiota could improve the prognosis of ischemic stroke.

## 1 Introduction

Stroke is the second leading cause of the global disease burden, with approximately 70%–80% of cases being ischemic strokes (1). Early prevention is currently the main strategy to reduce the disability and death caused by ischemic stroke. After stroke, early reperfusion therapy within an appropriate time window is crucial. Unfortunately, a significant portion of patients are unable to receive timely reperfusion, necessitating urgent exploration of brain protection and strategies to improve the functional prognosis after stroke (2). Recently, increasing evidence has suggested a close connection between the gut microbiota and ischemic stroke (3). Gut microbiota-targeted strategies to improve stroke outcomes have already been proposed (2).

Several clinical observational studies (2–4), and even some causal inference studies (5, 6), have confirmed the relationship between the gut microbiota composition and stroke risk and its associated risk factors. In addition, various risk factors in stroke-prone individuals and post-stroke conditions, such as post-stroke infection, depression, and behavior changes, could affect the balance of the gut microbiota (3, 7, 8). However, the association between gut microbiota changes and post-stroke prognosis lacks clear and high-quality evidence. Animal studies have indicated that improving the infarct volume and functional deficit is possible through the transplantation of fecal or microbial communities known to produce short-chain fatty acids (SCFAs) in mice (9, 10). The mechanisms underlying this effect are still unclear, but current research supports the bidirectional connection between the gut microbiota and the brain through the gut–brain axis. Bacterial compounds or metabolites may enter the bloodstream and cross the blood–brain barrier (2). Furthermore, some clinical trials have found a decrease in the incidence of systemic infection and a shorter length of hospitalization in stroke patients using probiotics such as *Bifidobacterium* or lactic acid bacteria (11, 12). However, these studies had limited sample sizes and multiple confounding factors and lacked clear assessment of functional prognosis. Moreover, post-stroke patients already experience gut microbiota dysbiosis, making it difficult to exclude the influence of reverse causality (3). Therefore, strong evidence is still required to support gut microbiota-targeted strategies to improve stroke outcomes.

Mendelian randomization (MR) uses genetic variations to assess the impact of different exposures on the outcomes. Since genetic variations are randomly allocated during meiosis and then are not affected by confounding factors or reverse causality, MR is often used to explore the causal relationships between two phenotypes (13, 14). Previous studies have primarily used MR analysis to explore the relationship between the gut microbiota and the risk of stroke occurrence, but the relationship with stroke functional prognosis has not yet been investigated (5, 6). With the recent publication of genome-wide association studies (GWAS) conducted by the Genetics of Ischemic Stroke Functional Outcome (GISCOME) network and the Vitamin Intervention for Stroke Prevention (VISP), researchers now have the opportunity to explore the causal relationship between these factors (15). In this study, we conducted a two-sample MR analysis using publicly available GWAS data to investigate the causal association between the gut microbiota and the functional outcome after ischemic stroke. In addition, to explore potential biological mechanisms, we performed enrichment analysis using identified causal instrumental variables (IVs).

## 2 Materials and methods

### 2.1 Mendelian randomization design

**Figure 1:**
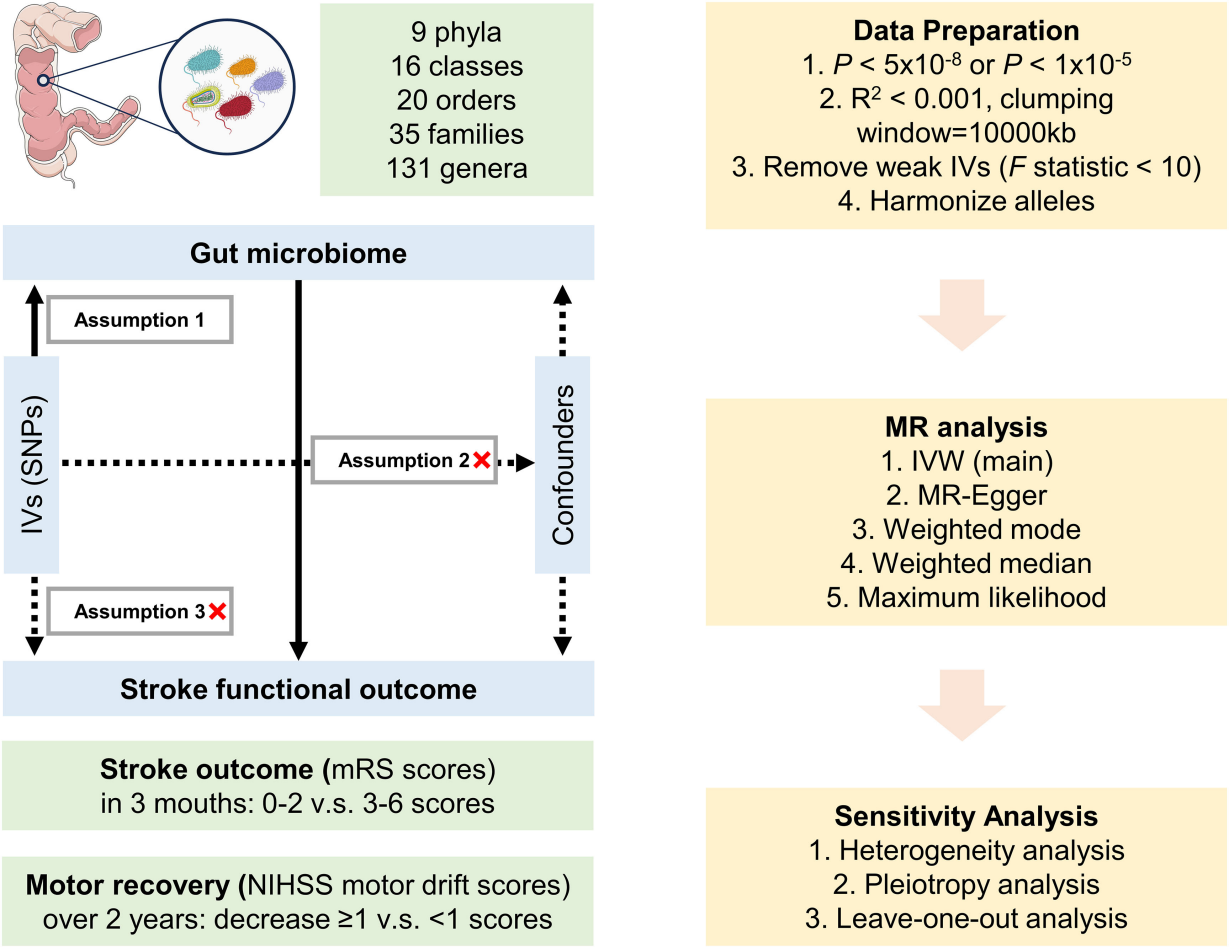
Design of the Mendelian randomization study. Parts of the figure were drawn using pictures from Servier Medical Art, by Servier, licensed under a Creative Commons Attribution 3.0 Unported License (https://creativecommons.org/licenses/by/3.0/).

This study utilized publicly available summary-level datasets sourced from original studies that have obtained approval from relevant ethics committees and informed consent from participants. **Figure 1** presents a visual representation of the study design. In this particular investigation, genetic variants were employed as IVs for the purpose of conducting MR analysis. The credibility of our MR study rests upon three fundamental assumptions: 1) the relevance assumption, which asserts a strong correlation between the genetic variants and the exposure; 2) the independence assumption, indicating that genetic variants have no association with the confounding factors; and 3) the exclusion restrictions assumption, which states that genetic variants solely influence the outcome via the exposure and not through alternative pathways.

### 2.2 Data sources

The summary statistics for the phenotypes of the gut microbiota and the functional outcome after ischemic stroke were obtained from previous GWAS. The selection criteria for GWAS were based on the following two points: 1) publicly available GWAS, and 2) for the same phenotype, only including GWAS with the largest sample sizes. The original paper provides detailed information regarding the recruitment criteria of the population and the quality control of genetic data.

Summary-level data for gut microbiota were extracted from the largest meta-analysis GWAS to date conducted by the MiBioGen consortium (16). A total of 18,340 individuals from 24 cohorts were included in this GWAS study, with the majority having European ancestry (72.3%). The microbial composition of distinct cohorts was profiled by targeting three different variable regions of the 16S rRNA gene: V4, V3–V4, and V1–V2. All microbiome datasets were standardized by rarefying them to 10,000 reads per cohort. A mapping analysis using microbiota quantitative trait loci was performed to identify the host genetic variants that corresponded to the genetic loci linked to the abundance levels of the bacterial taxa. Finally, a total of 211 bacterial taxa (9 phyla, 16 classes, 20 orders, 35 families, and 131 genera) were included.

Two phenotypes for functional outcome after ischemic stroke were included. The first phenotype used the modified Rankin scale (mRS) to assess the overall stroke outcome. The GWAS summary statistics for overall stroke outcome were obtained from GISCOME, which included 6,021 patients with ischemic stroke of European ancestry from 12 studies (17). The participants included were aged 18 years and older from locations in Europe, the United States, and Australia. Ischemic stroke is typically diagnosed based on clinical symptoms and imaging examinations, and the severity of stroke is assessed using the NIH Stroke Scale (NIHSS). According to the TOAST classification, ischemic stroke in GISCOME is divided into cardioembolic (31.7%), large artery atherosclerotic (17.9%), small vessel disease (19.2%), and other/undetermined (30.2%). The functional outcome after ischemic stroke was evaluated at 90 days using the mRS, which ranges from 0 (no symptoms) to 6 (death). The GISCOME study provided the GWAS data for two dichotomous variables, which compared scores of 0–2 *versus* 3–6 and scores of 0–1 *versus* 2–6, as well as a continuous variable known as the “mRS at 3 months” phenotype. A favorable functional outcome after ischemic stroke, indicating a lower mRS score of 0–2, was observed in a total of 3,741 cases. Conversely, a poor functional outcome, represented by a higher mRS score of 3–6, was observed in 2,280 subjects. We utilized the GWAS summary data for the comparison of mRS 0–2 *versus* 3–6, which were adjusted for age, sex, ancestry, and baseline NIHSS score, in accordance with previous studies (18, 19). Another phenotype used the motor drift subscores of the NIHSS as a measurement of motor recovery post-stroke. The GWAS summary statistics were collected from VISP, which encompassed 488 patients exhibiting weakness in an arm or a leg, defined as a motor drift time greater than zero on the NIHSS during randomization. Patients who had an incident recurrent stroke during the trial were excluded. Among the included patients, 73% were of European ancestry, with an average age of 66 years. The NIHSS subscores 5A/5B and 6A/6B assess the degree of limb weakness in the upper and lower extremities, also known as “‘drift.” The GWAS employed logistic regression models with generalized estimating equations to analyze the repeated NIHSS motor score measurements across six time points spanning a 24-month period. The primary outcome was dichotomized based on a decrease in the initial motor drift by ≥1 vs. <1 for each follow-up period (20). Specific details of the population characteristics for each GWAS can be found in the original publications (17, 20).

### 2.3 Selection of instrumental variables

The genetic IVs, typically single nucleotide polymorphisms (SNPs), for the gut microbiota and the functional outcome after ischemic stroke were extracted based on the same following selection criteria:

1) Considering that strict threshold criteria (*p* < 5.0 × 10^−8^) would result in some microbial genera having no available IVs, as well as only one SNP (*rs1842681*) being available for the overall stroke outcome phenotype, we identified IVs at the locus-wide significance threshold (*p* < 1.0 × 10^−5^) (21).

2) Genetic variants were clumped at a linkage disequilibrium threshold of *R^2^* < 0.001 within ±10,000 kb distance using the 1000 Genomes European reference panel.

3) To ensure that the effects of the SNPs on the exposure align with the same allele effects on the outcome, we excluded palindromic SNPs from the IVs.

4) We used the *F*-statistic [*F* = (*β*/se)^2^] to measure the strength of the relationship between the genetic variants and the exposure variable. A higher *F*-statistic indicates a stronger instrument. In this study, SNPs with *F*-statistics <10 were considered weak IVs and were then excluded from the MR analysis (19).

### 2.4 Statistical analyses

In this study, a two-sample MR analysis was performed to evaluate the causal links between 211 microbial taxa and the functional outcome after ischemic stroke. To detect causal effects between the exposure and outcomes, five distinct methods were employed: inverse variance weighted (IVW), MR-Egger, weighted median, weighted mode, and maximum likelihood. Specifically, by employing a meta-analysis approach and the Wald estimates for each SNP, the IVW method aimed to derive an aggregated estimate of the effect of the gut microbiota on the functional outcome after ischemic stroke. It is important to note that the IVW results would be unbiased only if horizontal pleiotropy was absent (22). Based on the assumption of instrument strength independent of direct effect (InSIDE), MR-Egger regression allows for the assessment of pleiotropy through the intercept term. If the intercept term is equal to zero, the results of the MR Egger regression will be consistent with the IVW method, indicating the absence of horizontal pleiotropy (23). The weighted median method provides a means for the unbiased estimation of causal effects, even in conditions where as many as 50% of the IVs are invalid (24). The weighted mode method estimates the causal effect by focusing on the largest cluster of valid IVs. Even in conditions where there are invalid instruments, the weighted mode method maintains consistency as long as the highest number of similar individual instrument causal effect estimates is obtained from the valid instruments (25). Under the assumption of no heterogeneity and horizontal pleiotropy, the maximum likelihood method exhibits a resemblance to IVW. The results will be unbiased and the standard errors will be smaller than IVW if the hypotheses are satisfied (26). A Bonferroni correction was performed to evaluate multiple comparisons, with the threshold set at 0.05 divided by the number of each bacterial taxon to declare significant association. A *p* < 0.05 was considered to have a suggestive association (21). A reverse causality analysis was conducted to evaluate the reverse causal association between the functional outcome and the bacterial taxa.

In the sensitivity analyses, Cochran’s *Q* statistics were calculated to detect heterogeneity through the IVW and MR-Egger methods (27). MR-Egger regression and Mendelian randomization pleiotropy residual sum and outlier (MR-PRESSO) were performed to evaluate the potential pleiotropy (23, 28). The MR-PRESSO method was utilized to identify and address any potential outliers that might indicate pleiotropy bias, while also correcting for horizontal pleiotropy. Furthermore, leave-one-out sensitivity analyses were performed to ensure the stability of the causal effect estimates. This involved systematically excluding each SNP from the instrumental variables to examine the potential presence of outliers.

### 2.5 Enrichment analysis

After the MR analysis and sensitivity analyses, the potential biological mechanisms underlying the causal effect of the gut microbiota on the functional outcome after ischemic stroke were further investigated through the Metascape platform, an automated meta-analysis tool to understand biological pathways (29). Enrichment analysis was carried out with the Gene Ontology (GO) biological processes, Reactome gene sets, and the Kyoto Encyclopedia of Genes and Genomes (KEGG) pathway using the nearest genes identified from the causal SNPs (30).

R software (version 4.1.3) was utilized to conduct the statistical analysis. The package “TwoSampleMR” (version 0.5.10) was employed to perform the MR analysis, MR-Egger regression, and Cochran’s *Q* test, while the package “MRPRESSO” (version 1.0) was used for the MR-PRESSO analysis and the package “vautils” used for the identification of the nearest genes.

## 3 Results

Details on the genetic variables for the gut microbiota and the functional outcome after ischemic stroke in the MR analyses are presented in **Supplementary Tables S1** and **S2**. For the selection process of IVs, a collection of 2,213 SNPs was employed as the IVs across 211 bacterial taxa. In the reverse MR analysis, 12 and 16 SNPs were identified for overall stroke outcome and motor recovery, respectively. All of the IVs utilized in the MR analyses exhibited *F*-statistics exceeding 10, suggesting the absence of weak IVs.

### 3.1 Causal effects of the gut microbiota on overall stroke outcome

**Figure 2:**
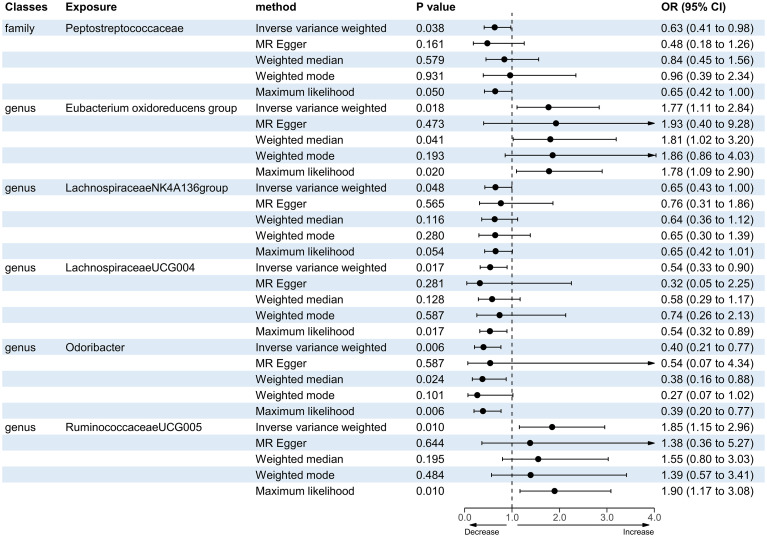
Forest plot of the significant Mendelian randomization (MR) results of the causal relationship between the gut microbiota and overall stroke outcome. *OR*, odds ratio; 95% CI, 95% confidence interval.

**Figure 3:**
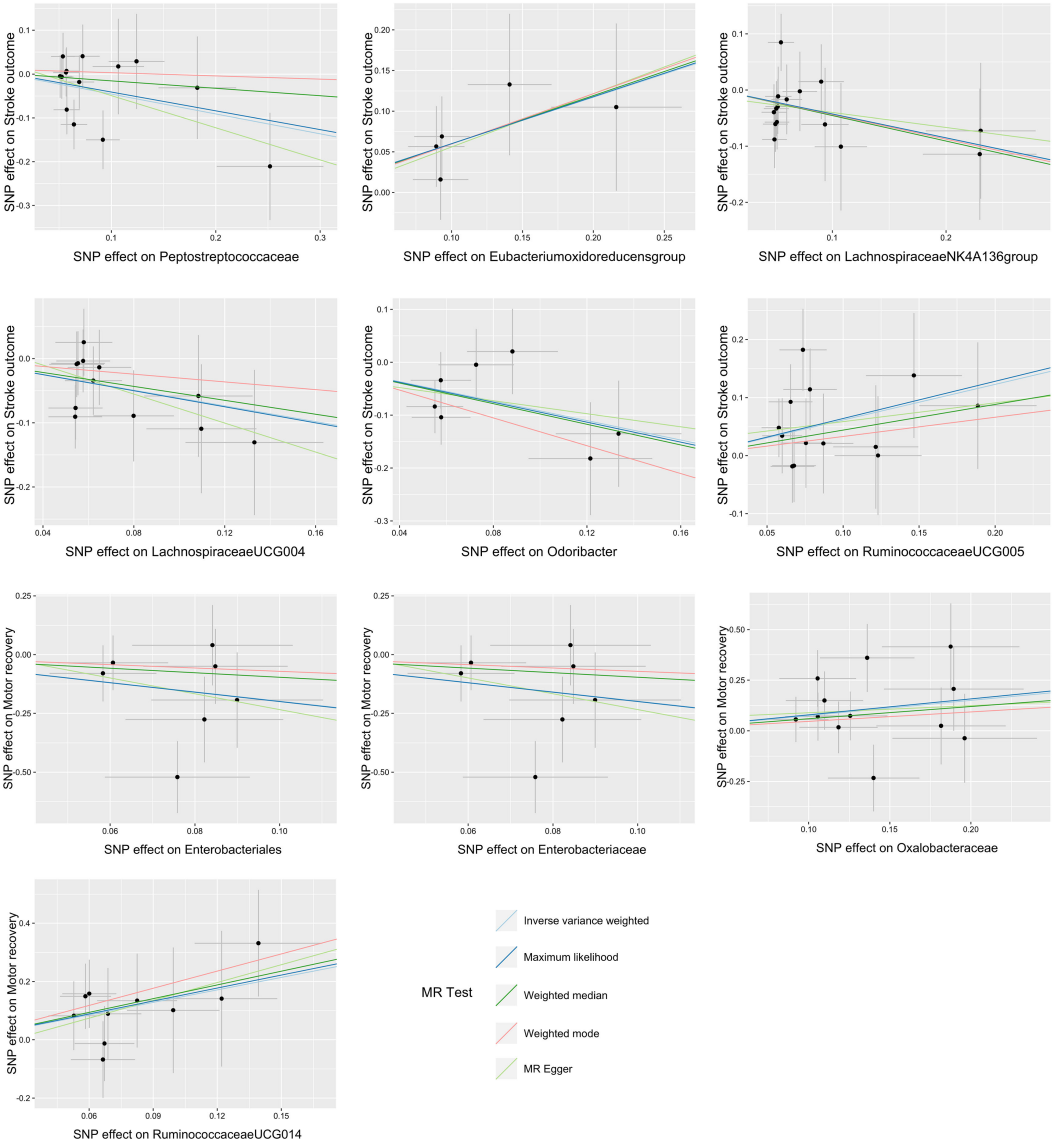
Scatter plots of the causal association between the gut microbiota and functional outcome after ischemic stroke. The Mendelian randomization (MR) results for the order Enterobacteriales and the family Enterobacteriaceae were consistent, resulting in identical images.

A total of 12 bacterial taxa were identified to be suggestively associated with overall stroke outcome using the IVW analysis, as shown in **Supplementary Table S3**. Class Verrucomicrobiae, order Verrucomicrobiales, family Verrucomicrobiaceae, and the genera *Akkermansia*, *LachnospiraceaeND3007 group*, and *RuminococcaceaeUCG013* were excluded from the positive results due to MR-Egger showing a different effect direction from the IVW methods. Finally, six bacterial taxa showed consistent directionality across all MR analysis methods, as shown in **Figure 2**. Specifically, the IVW analysis suggested that the genetically predicted family Peptostreptococcaceae (OR = 0.63, 95% CI = 0.41–0.98, *p* = 0.038) and the genus *LachnospiraceaeNK4A136 group* (OR = 0.65, 95% CI = 0.43–1.00, *p* = 0.048) had a suggestive association with favorable functional outcome. The genus *LachnospiraceaeUCG004* was suggestively associated with favorable functional outcome using the IVW method (OR = 0.54, 95% CI = 0.33–0.90, *p* = 0.017) and the maximum likelihood method (OR = 0.54, 95% CI = 0.32–0.89, *p* = 0.017). The genus *Odoribacter* also presented a suggestive association with favorable functional outcome using the IVW method (OR = 0.40, 95% CI = 0.21–0.77, *p* = 0.006), the weighted median method (OR = 0.38, 95% CI = 0.16–0.88, *p* = 0.024), and the maximum likelihood method (OR = 0.39, 95% CI = 0.20–0.77, *p* = 0.006). On the contrary, the genus *Eubacterium oxidoreducens group* was associated with unfavorable functional outcome according to the IVW method (OR = 1.77, 95% CI = 1.11–2.84, *p* = 0.018), the weighted median method (OR = 1.81, 95% CI = 1.02–3.20, *p* = 0.041), and the maximum likelihood method (OR = 1.78, 95% CI = 1.09–2.90, *p* = 0.020). The genus *RuminococcaceaeUCG005* also presented a suggestive association with unfavorable functional outcome according to the IVW method (OR = 1.85, 95% CI = 1.15–2.96, *p* = 0.010) and the maximum likelihood method (OR = 1.90, 95% CI = 1.17–3.08, *p* = 0.010). None of the results were significant after Bonferroni multiple test correction. **Figure 3** displays the scatter plot illustrating the causal effect of the gut microbiome on overall stroke outcome.

### 3.2 Causal effects of the gut microbiota on motor recovery post-stroke

**Figure 4:**
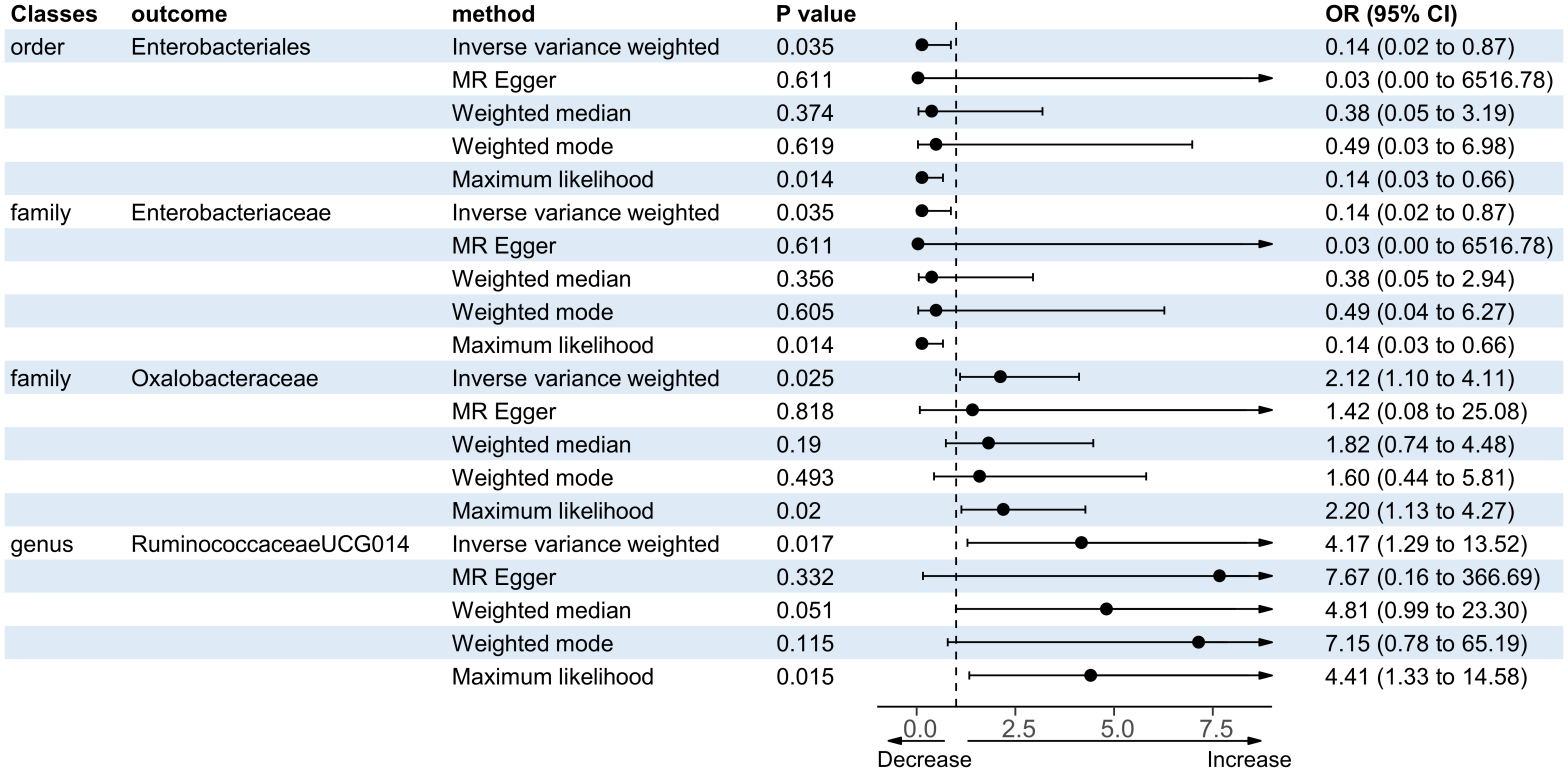
Forest plot of the significant Mendelian randomization (MR) results of the causal relationship between the gut microbiota and motor recovery. *OR*, odds ratio; 95% CI, 95% confidence interval.

**Figure 5:**
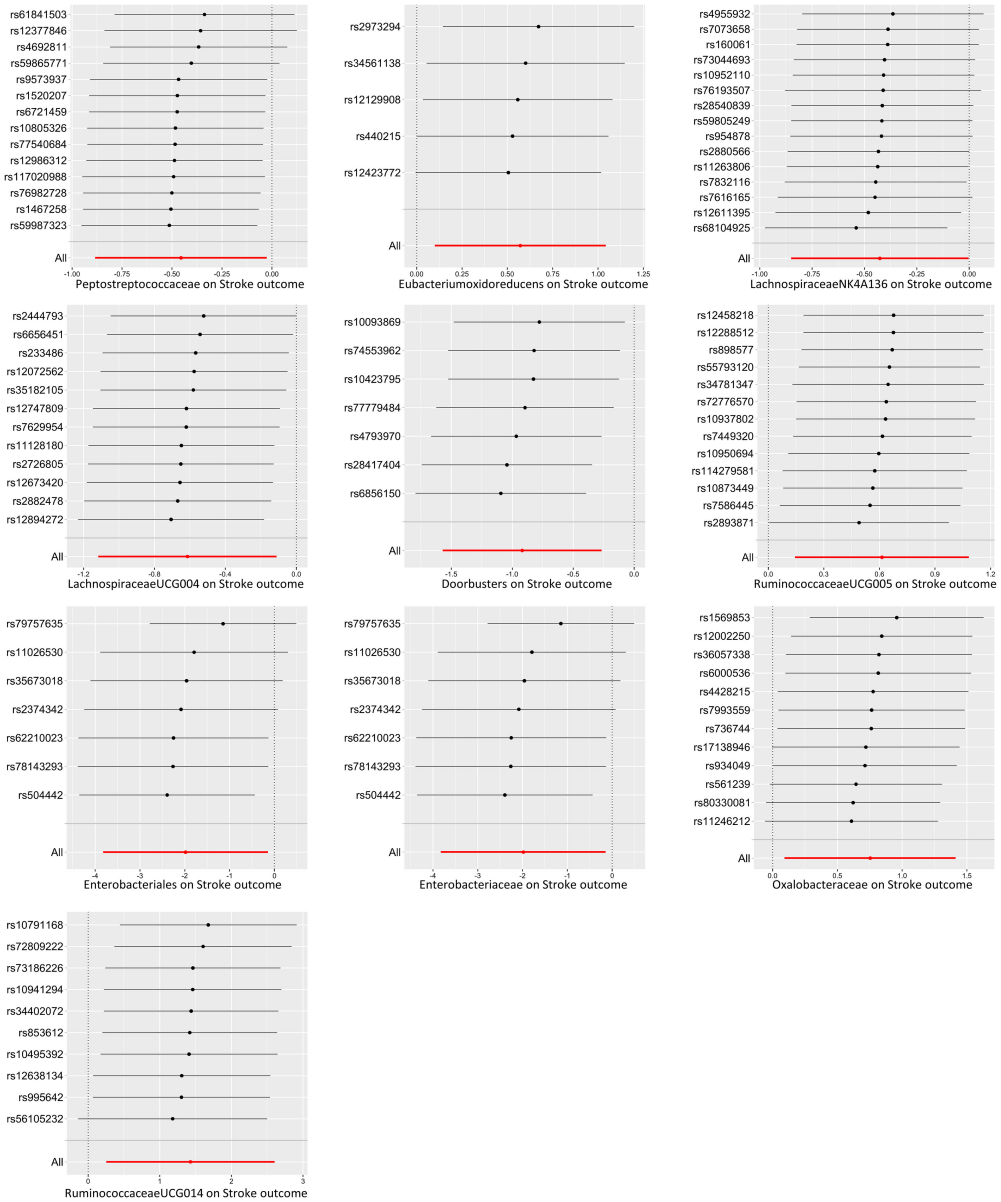
Leave-one-out plots of the causal association between the gut microbiota and functional outcome after ischemic stroke. The Mendelian randomization (MR) results for the order Enterobacteriales and the family Enterobacteriaceae were consistent, resulting in identical images.

There were five bacterial taxa identified to be suggestively associated with motor recovery using the IVW analysis, as shown in **Supplementary Table S3**. The family Peptostreptococcaceae was excluded from the positive results due to MR-Egger showing a different effect direction from the IVW methods. Finally, four bacterial taxa showed consistent directionality across all MR analysis methods, as shown in **Figure 4**. Specifically, the IVW analysis suggested that the genetically predicted family Oxalobacteraceae (OR = 2.12, 95% CI = 1.10–4.11, *p* = 0.025) and the genus *RuminococcaceaeUCG014* (OR = 4.17, 95% CI = 1.29–13.52, *p* = 0.017) had a suggestive association with motor recovery. On the contrary, the order Enterobacteriales (OR = 0.14, 95% CI = 0.02–0.87, *p* = 0.035) and the family Enterobacteriaceae (OR = 0.14, 95% CI = 0.02–0.87, *p* = 0.035) were associated with motor weakness according to the IVW method. These positive associations were also replicated using the maximum likelihood method. None of the results were significant after Bonferroni multiple test correction. **Figure 3** displays the scatter plot illustrating the causal effect of the gut microbiome on motor recovery post-stroke. It is important to note that, since the IVs (SNPs) and their effect values for the order Enterobacteriales and the family Enterobacteriaceae obtained from the original GWAS data were consistent (**Supplementary Table S1**), the MR results based on the IVW method (**Figure 2**), the scatter plots (**Figure 3**), and the leave-one-out analysis results (**Figure 5**) for these two exposures were identical. This could be due to the consistent GWAS results for some adjacent bacterial taxon phenotypes, a phenomenon that has also been observed in some previous MR studies (31, 32).

### 3.3 Sensitivity analysis

In the sensitivity analyses, the Cochran’s IVW (*p* range, 0.06–0.93) and the MR-Egger *Q* test (*p* range, 0.12–0.90) results indicated the absence of a significant heterogeneity for the positive findings (**Supplementary Table S4**). Furthermore, the results of the MR-Egger regression intercept analysis (*p* range, 0.54–0.92) and the MR-PRESSO analysis (*p* range, 0.14–0.97) revealed no significant horizontal pleiotropy (**Supplementary Tables S5**, **S6**). The leave-one-out analysis revealed that not one SNP was responsible for the causal effects of the gut microbiome on the functional outcome after ischemic stroke (overall stroke outcome and motor recovery), as shown in **Figure 5**. In the reverse MR analysis, no significant causal effect of functional outcome after ischemic stroke (overall stroke outcome and motor recovery) was found on the bacterial taxa that were found to be causally associated with functional outcome in the forward MR analysis (**Supplementary Table S7**). No heterogeneity statistics and horizontal pleiotropy were identified (**Supplementary Tables S8**-**S10**).

### 3.4 Enrichment analysis

**Figure 6:**
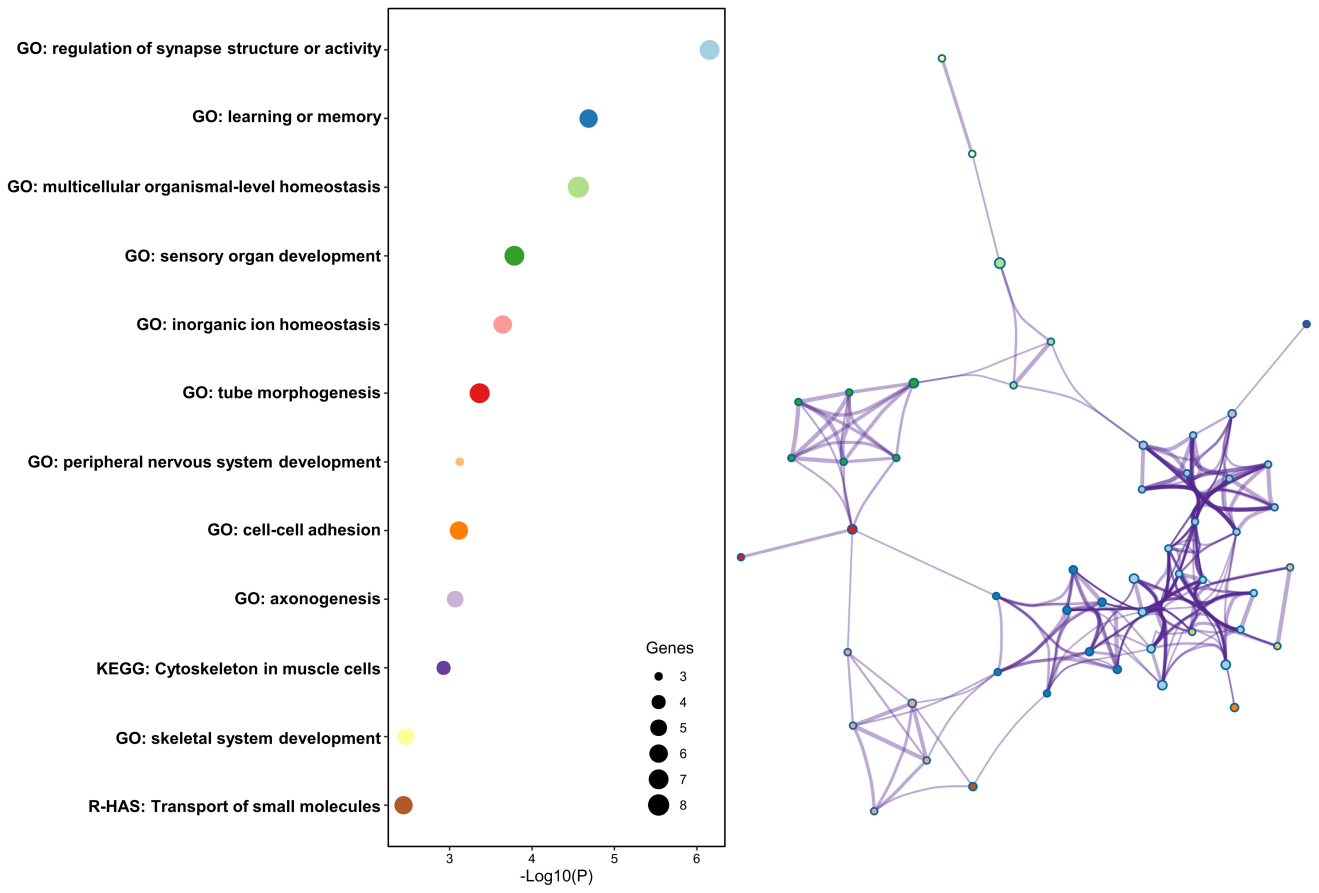
Results of the enrichment analysis.

Finally, 63 genes were identified from the 121 causal SNPs identified from the MR analysis (**Supplementary Table S11**). The relevant pathways were mainly enriched in the regulation of synapse structure or activity, learning or memory, multicellular organismal-level homeostasis, sensory organ development, and inorganic ion homeostasis pathway in the GO analysis, the cytoskeleton in muscle cells pathway in the KEGG analysis, and the transport of small molecules pathway in the Reactome gene set analysis (**Figure 6**).

## 4 Discussion

In this study, MR analysis was conducted to investigate the causal effect of the gut microbiota on the functional outcome after ischemic stroke (overall stroke outcome and motor recovery) and potential biological mechanisms. The results revealed a total of six bacterial taxa observed to be causally linked to the overall stroke outcome based on mRS measurement and four bacterial taxa associated with motor recovery based on NIHSS motor drift measurement. Among them, the family Peptostreptococcaceae and the genera *LachnospiraceaeNK4A136 group*, *LachnospiraceaeUCG004*, and *Odoribacter* showed a suggestive association with favorable functional outcome, while the genera *Eubacterium* (*oxidoreducens group*) and *RuminococcaceaeUCG005* were associated with unfavorable functional outcome. The family Oxalobacteraceae and the genus *RuminococcaceaeUCG014* were associated with motor recovery, while the order Enterobacteriales and the family Enterobacteriaceae were linked to motor weakness. Furthermore, the enrichment analysis suggested that the regulation of synapse structure or activity could be involved in mediating their causal relationship. Unlike previous MR studies, our research simultaneously explored the causal relationships between the gut microbiota and both the overall stroke prognosis and motor recovery, emphasizing the close association between SCFA-producing bacteria and stroke outcomes and revealing the potential mediating mechanisms through gene enrichment analysis.

Previous studies have shown that changes in the gut microbiota composition and function are closely associated with the severity of stroke and disability post-stroke (2). Animal studies have demonstrated that, compared with mice with normal gut microbiota, germ-free mice transplanted with the gut microbiota of stroke-affected mice or humans exhibited larger infarct volumes and more severe neurological deficits after acute middle cerebral artery occlusion (33, 34). In recent clinical observational studies, a significant reduction of *Faecalibacterium* and *Faecalibacterium prausnitzii* was observed in the non-minor stroke and 3-month poor prognosis groups (35). A study summarizing 14 clinical studies revealed that post-stroke patients showed 62 upregulated and 29 downregulated microbial taxa, along with a reduced gut microbiome diversity compared with healthy controls (3). In fact, patients with ischemic stroke and severe stroke outcomes had a lower abundance of SCFA-producing bacteria (3, 4, 36).

Most studies have observed an upregulation of Lachnospiraceae in stroke patients (3, 35). In this study, it was revealed that the elevation of the genera *LachnospiraceaeNK4A136 group* and *LachnospiraceaeUCG004* could promote stroke functional recovery. These findings are supported by previous research. An observational study indicated that the levels of Lachnospiraceae in post-stroke patients were negatively correlated with the mRS score (*R* = −0.24) (37). Lachnospiraceae belongs to butyrate-producing bacteria. Recent animal studies have demonstrated that butyrate, a SCFA, significantly reduces the infarct volume after stroke and improves the prognosis and cognitive function, possibly by promoting angiogenesis and blood–brain barrier integrity and by reducing leukocyte infiltration (38, 39). Interestingly, an MR analysis indicated that *LachnospiraceaeNK4A136 group* could potentially lower the risk of both large artery and small vessel strokes (5). Lachnospiraceae may be a key bacterial taxon in the prevention of stroke occurrence and in the improvement of stroke prognosis. These results suggest that changes in the gut microbiota composition, particularly in Lachnospiraceae, could be a compensatory response for stroke. We hypothesize that Lachnospiraceae could reduce the occurrence of stroke and that, once a stroke occurs, its levels may be increased to promote stroke functional recovery. Appropriately increasing the proportion of Lachnospiraceae in the gut of stroke patients could potentially improve the stroke functional prognosis. This study also suggests the potential contribution of *Odoribacter* to a favorable stroke prognosis. Consistent with these findings, a previous study found a correlation between increased *Odoribacter* levels and favorable outcomes in acute ischemic stroke patients with H-type hypertension (3). Furthermore, this study revealed that another common SCFA-producing bacterium, *RuminococcaceaeUCG014*, could promote motor recovery. In summary, this study reveals a potential impact of microbial taxa associated with SCFA production on stroke prognosis. In fact, several animal experiments have used SCFAs to treat post-stroke mice through supplementation with SCFAs or through fecal transplantation with SCFA-producing microbiota and have found improved post-stroke recovery (9, 10). In a case–control study including 140 patients with acute ischemic stroke, a poor functional outcome at 3 months was associated with reduced levels of SCFAs, especially acetate (36). This study strengthens this conclusion. Treatment strategies focused on modulating the gut microbiota and SCFAs could potentially improve the clinical outcomes of patients (40). However, it remains unclear whether these microbiota changes are specifically linked to stroke. A significant amount of research indicates a connection between the reshaping of the gut microbiota metabolites—specifically microbiota-derived SCFAs—and the pathophysiology of various neurological diseases, including Alzheimer’s disease, multiple sclerosis, Parkinson’s disease, and amyotrophic lateral sclerosis (41).

However, this study also reveals a potential hindrance to stroke functional recovery posed by other common SCFA-producing bacteria, including *RuminococcaceaeUCG005* and *Eubacterium* (*oxidoreducens group*). Multiple studies have reported a higher prevalence of Ruminococcaceae and a lower prevalence of *Eubacterium* in post-stroke patients (3, 35). Various observational studies have identified 62 upregulated and 29 downregulated microbial taxa among stroke patients; however, the regulation of a certain taxon can vary, being either upregulated or downregulated, depending on the specific cohort or study (3). This implies that post-stroke changes in the gut microbiota may be a double-edged sword or a manifestation of the coexistence of both compensation and decompensation (42). Evidence supports dynamic changes in the gut microbiota following a stroke (43). One confounding factor to consider is the potential association between post-stroke infections and gut microbiota dysbiosis, which could influence stroke prognosis (44). Furthermore, in addition to SCFAs, other mechanisms such as immunomodulators or other metabolic pathways [e.g., trimethylamine-*N*-oxide (TMAO) and tryptophan metabolites] could be involved in the process of the gut microbiota regulating the prognosis of stroke. A previous study observed a larger infarct volume and more severe motor deficits in mice administered choline or TMAO before a stroke, or those receiving a transplant of a functional gut microbial choline utilization C (CutC) enzyme, which converts choline to trimethylamine and subsequently elevates the TMAO levels (45). Intestinal microorganisms influence the host immune system partly by producing metabolites that interact with host receptors and other target molecules (46). AMP-activated protein kinase mediators have been shown to play dual roles in modulating the gut microbiota and in regulating neuroinflammation and neuronal apoptosis following a stroke (47). In addition, in this study, Peptostreptococcaceae was found to be associated with a favorable functional outcome in stroke. However, a clinical observational study found elevated levels of Peptostreptococcaceae in patients with post-stroke depression, suggesting an association between Peptostreptococcaceae and poor prognosis (7). Further research is needed to clarify these inconsistent findings.

In addition to finding an association between SCFA-producing bacteria and stroke outcomes, we also discovered an association between Enterobacteriaceae and stroke outcomes. The results of this study suggest that an increase in Enterobacteriaceae in the gut could hinder motor recovery. A previous study has shown that the onset of ischemic stroke could quickly lead to intestinal ischemia, triggering excessive nitrate production through free radical reactions, in turn leading to dysbiosis of the gut microbiota characterized by an increase in Enterobacteriaceae. The proliferation of Enterobacteriaceae further exacerbates brain infarction by intensifying systemic inflammation, thereby acting as an independent risk factor for adverse primary outcomes in stroke patients (48). A recent study has highlighted the role of Enterobacteriaceae enrichment as a key mediator linking coronavirus disease 2019 (COVID-19) infection to poor stroke outcomes (44). Furthermore, another study revealed significantly higher levels of Enterobacteriaceae in the gut of patients with post-stroke cognitive impairment (39). Taking our findings together, we speculate that Enterobacteriaceae can not only impact cognitive function after stroke but also affect motor function after stroke. Research has shown the influence of Enterobacteriaceae on motor symptoms in patients with Parkinson’s disease (49). Further interventional studies are warranted to explore whether reducing the levels of Enterobacteriaceae in the gut could improve motor symptoms and muscle strength.

The mechanisms through which the gut microbiota influence the functional outcomes after stroke are not yet fully understood. Current hypotheses suggest that the metabolites produced by the gut microbiota, such as TMAO and SCFAs, impact brain function through various pathways (2, 3). In addition, the gut microbiota may indirectly affect brain function by modulating systemic inflammation (3, 50). Our research found that causal genes linking the gut microbiota and functional outcomes after stroke are mainly enriched in synaptic tissue regulation. This finding is supported by an animal experiment (9). Supplementation of SCFAs in the drinking water of male mice with stroke notably enhanced the restoration of motor function in the affected limb. Changes in the contralesional cortex connectivity induced by SCFAs were also observed, which were associated with alterations in the spine and synapse densities. Therefore, we speculate that the gut microbiota, especially those associated with SCFAs, could affect stroke prognosis by mediating synapse function.

Several limitations of this study are worth considering. Firstly, the GWAS data used in this study primarily came from European participants, which limits the generalizability of the results. However, most studies on the relationship between gut microbiota alteration and stroke severity and outcomes have been conducted in China (3). This study extended the findings to European populations and discovered several consistent results. Secondly, the GWAS data using mRS as the assessment tool for stroke functional outcomes did not provide specific information on the different subtypes of functional prognoses (15). To address this limitation, we included motor recovery phenotypes based on NIHSS motor drift measurement. However, the evaluation of other functional outcomes such as post-stroke cognitive impairment or post-stroke depression is currently unavailable, which limits the interpretability of our results. Thirdly, as the publicly available GWAS data for stroke functional outcomes did not differentiate between different stroke subtypes, assessment of functional outcomes across stroke subtypes is currently not possible. Previous findings have suggested that the relationship between gut microbiota and stroke may vary across stroke subtypes. Fourthly, the MiBioGen meta-analysis exhibited a considerable degree of variability, with only nine taxa detected in over 95% of the samples, and it only allowed the detection of genetic data at the genus to the phylum level. This high variability could potentially impact the precision and reliability of the study findings (16). Fifthly, as the gut microbiome GWAS data we included only pertained to the bacteria present in the gut, we were unable to determine the causal relationship between viruses, fungi, archaea, or microbiota from other locations, such as the oral microbiome, and stroke outcomes. Previous studies have found that, compared with the sham group, mice with transient focal ischemia showed a decrease in the abundance of two viral taxa and an increase in the abundance of five viral taxa (51). An observational study that included 146 participants found that, compared with healthy controls, patients with ischemic stroke had higher diversity in their oral salivary microbiome, and some differential bacteria had predictive value for the severity and prognosis of ischemic stroke (52). Lastly, while our MR study suggests a potential causal relationship between the gut microbiota and the functional outcomes after ischemic stroke, it is important to note that Bonferroni correction is considered a conservative approach.

## 5 Conclusions

In summary, this MR study reveals the causal effect of the gut microbiota, especially the SCFA-producing microbiota, on the functional outcomes after ischemic stroke. In addition, Enterobacteriaceae in the gut could hinder motor recovery. The gut microbiota may influence stroke prognosis by modulating synaptic function. Future research should further explore the mechanisms and more clinical trials on gut microbiota-targeted strategies should be conducted to improve stroke outcomes.

## Data Availability

The original contributions presented in the study are included in the article/**Supplementary Material**. Further inquiries can be directed to the corresponding author.
